# Toxicology and Pharmacokinetics Study of Intradiscal Injection of Simvastatin in Rabbits

**DOI:** 10.3389/fphar.2021.582309

**Published:** 2021-04-23

**Authors:** Xiaodong Huang, Wei He, Weiheng Wang, Quanchun Fan, Xiaojian Ye, Zenghui Wu, Chia-Ying Lin

**Affiliations:** ^1^ Department of Orthopedics, The Third Affiliated Hospital of Guangzhou Medical University, Guangdong, China; ^2^ Department of Orthopaedic Surgery, College of Medicine, University of Cincinnati, Cincinnati, OH, United States; ^3^ Department of Orthopaedics, Shanghai Changzheng Hospital, Shanghai, China; ^4^ Department of Spine Surgery, Beijing Jishuitan Hospital, 4th Medical College of Peking University, Beijing, China; ^5^ Department of Orthopaedics, Fuzhou Second Hospital Affiliated to Xiamen University, Fujian, China

**Keywords:** intervertebral disc, simvastatin, pharmacokinetics, intradiscal injection, toxicity

## Abstract

To test the pharmacokinetics and toxicology of whole organs and tissues after intradiscal injection of simvastatin in rabbits. To provide the information needed to support human clinical trials. Twelve male and twelve female rabbits were randomly divided into four groups: control group (0 mg/ml), low dose group (0.1 mg/ml), medium dose group (1 mg/ml) and high dose group (10 mg/ml). Simvastatin at different concentrations of 10 μl was injected into L3/4, L4/5 and L5/6 intervertebral discs in each group. Poly (ethylene glycol) -poly (lactic-co-glycolic acid) -poly (ethylene glycol) (PEG-PLGA-PEG) polymer as the drug carrier. The pharmacokinetics of blood samples were measured by LC-MS/MS. Cerebrospinal fluid was obtained and the drug concentration was measured. Blood routine, blood biochemistry and urine of all animals were analyzed and evaluated. The heart, kidney, liver and spleen of each animal were observed and weighed. The intervertebral disc tissues were stained with hematoxylin and hematoxylin (H&E), and then qualitatively analyzed by optical microscopy. 28 days after intradiscal injection of simvastatin, 28 days after simvastatin intradiscal injection, there was no significant difference between the weight, food residue, blood routine, blood biochemistry, urine routine results and the weight of each organ in the four groups (*p* > 0.05). The serum concentration of simvastatin is lower than the lowest measurable concentration. The histological score of the intervertebral disc in the high-dose group was significantly higher than that in the other three groups at 28 days (*p* < 0.05). Three doses of simvastatin were injected into male and female animals respectively, showing no toxic effects. Microscopic histological evaluation of the intervertebral disc showed that the high dose group (10 mg/ml) had damage to the intervertebral disc tissue.

## Introduction

Lower back pain (LBP) is a worldwide epidemic disease (Murray et al., 2013). About 80% of people have experienced LBP at some or a long time in their lives (Andersson, 1999). If the course of disease exceeds three months, it is called chronic low back pain. In the United States, LBP is one of the most frequently used opioids other than tumors (Andersson, 1981; Ringwalt et al., 2014). According to statistics, the annual cost of back pain in the United States is about 200 billion US dollars (Dieleman et al., 2016) LBP not only greatly reduces people's quality of life, but also takes up a large part of medical resources. The main cause of LBP is the abnormal growth of nerve fibers into intervertebral discs, ligaments, articular processes, and other joints, causing pain below the costal margin and above the wrinkles below the hip. The main cause of LBP is from pain caused by degenerative disc (Adams, 2004), which accounts for more than 40% of LBP. Non-specific LBP caused by disc disease is called discogenic low back pain (DLBP).

The intervertebral disc is composed of an outer annulus fiberous, an inner gel-like center-nucleus pulposus, and cartilage end plates at both ends (Urban and Roberts, 2003), which are spinal cord remnants (McCann et al., 2012). It is the largest avascular tissue in the body. The nutrition of the intervertebral disc is mainly provided by the diffusion of the cartilage endplate. The annulus fiberous is composed of several layers of fibers, and the annulus fiberous is mainly composed of type I and type II collagen. Type I collagen is mainly concentrated in the outer layer of the annulus fiberous to provide greater strength. Type II collagen is mainly distributed in the inner layer of the annulus fiberous, maintaining the position and shape of the nucleus pulposus, and eventually migrating to the nucleus pulposus. The nucleus pulposus is a milky white translucent gelatinous body, rich in moisture and elasticity. It is the core of the intervertebral disc and acts as a shock absorber, absorbing the stress impact from the upper and lower end plates. The intervertebral disc contains an elastic nucleus pulposus, which helps to distribute the pressure uniformly in the intervertebral disc and avoid stress concentration, which may cause damage to the upper and lower end plates and the vertebral body and cause disc degeneration. The rigid end plate can withstand axial compressive forces and serves as the main channel for the nutritional supply of the disc.

With age, irreversible degeneration of the intervertebral disc occurs, the strength of the annulus fibrous decreases, the number of cells in the nucleus pulposus decreases, the elasticity decreases, and the cartilage endplate fragility increases. The clinical features of intervertebral disc degeneration include decreased intervertebral space height and decreased intervertebral disc water content. The factors of intervertebral disc degeneration are complex and multifactorial (Adams and Roughley, 2006). Mechanical stress, trauma, malnutrition, intravertebral disc inflammation, and aging are the main causes of disc degeneration (Adams and Roughley, 2006; Risbud and Shapiro, 2014). The risk factors of intervertebral disc degeneration have been extensively and thoroughly studied. When the disc is degenerated, the first matrix changes occur in the center of the nucleus pulposus, including the fragmentation of proteoglycan, followed by a decrease in proteoglycan and water content and a decrease in cell number (Buckwalter, 1995). Studies have shown that the role of proteoglycans in cartilage end plates is to regulate fluid balance in the disc (Roberts et al., 1996). When the disc is degenerated, the decrease of proteoglycan in the cartilage endplate will cause the decrease of proteoglycan in the nucleus pulposus. In addition, a reduction in lumbar blood flow reduces the ability of endplate cartilage to transport nutrients. Atherosclerosis and arterial calcification can cause lumbar artery blood flow to decrease, increase the possibility of disc degeneration, and eventually cause intervertebral DLBP (Kauppila et al., 1997). Intervertebral disc degeneration is closely related to intervertebral DLBP (Adams, 2004).

Recent studies have shown that bone morphogenetic proteins (BMP-2, -5, -6, -8, -9, and -14) play a crucial role in disc degeneration and cartilage endplate formation (Reddi, 1994; DiLeone et al., 1997; Sekiya et al., 2001; Rickert, 2008; Snelling et al., 2010). Studies have shown that statin cholesterol-lowering drugs that people take every day can stimulate bone formation by up-regulating the BMP-2 pathway (Mundy et al., 1999) simvastatin is an inhibitor of HMG-CoA reductase and a traditional inexpensive lipid-lowering drug. Simvastatin has been widely used as a drug for lowering blood lipids and preventing cardiovascular diseases, and its safety and cost in long-term clinical use have been widely recognized and accepted. Recent studies have shown that simvastatin can regulate bone and cartilage metabolism by up-regulating BMP-2A expression (Zhang and Lin, 2008). The effects and mechanisms of statins on lipid reduction have been widely studied and recognized, but their effects on bone and cartilage and their correlation have not been fully studied. Previous studies have shown that simvastatin can promote the proliferation of nucleus pulposus cells and the secretion of extracellular matrix *in vitro*. *In vivo* experiments of rat tail discs have also confirmed the effectiveness of simvastatin disc injection for disc degeneration, but its dose effect and toxicological effects have not been fully verified. At present, oral simvastatin has been recognized as very safe and reliable. The clinical application of simvastatin for the treatment of bone-related diseases requires a higher clinical dose than lipid-lowering, which will increase the statin-related side effects (Oryan et al., 2015), plus intervertebral discs. It is a relatively closed organ with relatively slow nutrient exchange. Therefore, the safety and pharmacokinetics of simvastatin through local injection of high concentration in the intervertebral disc are still unknown, which is related to the clinical application prospect of simvastatin.

Therefore, this study intends to conduct animal experiments on the pharmacokinetics and toxicological studies of simvastatin injected into the intervertebral disc for 28 days, in order to provide theoretical and experimental foundation for the clinical application of simvastatin.

## Methods and Materials

### Experimental Animals

Fifteen male and 12 female 6-month-old New Zealand rabbits (3–3.5 kg) were purchased from Qingdao Kangda Biotechnology Co., Ltd. Each cage (815 mm × 500 mm × 340 mm) holds one rabbit. These rabbits are housed in a specific room. The air filtration rate is 10–20 air changes per hour; the temperature is 20°–26°C. The humidity is 40–70%, and the fluorescent light is dark for 12 h (08:00–20:00) and then 12 h every day. These rabbits were housed in an environment where they could eat and drink freely, and adapted to the environment for at least 2 weeks before surgery. All animal operations in this experiment were approved by the Animal Ethics Committee of Naval Military Medical University.

### Animal Grouping

Based on body weight, a total of 12 male rabbits and 12 female rabbits were assigned to the treatment group by randomization in the BioBook system (IDBS). According to Table 1, 12 male rabbits and 12 female rabbits were used. Animals were evenly divided into four groups: the vehicle control group was injected with hydrogel + simvastatin (0 mg/ml, Control group), and the low-dose group was injected with hydrogel + simvastatin (0.1 mg/ml, Low dose group), The middle-dose group is injected hydrogel + simvastatin (1 mg/ml, Medium dose group), the high-dose group is injected hydrogel + simvastatin (10 mg/ml, High dose group, *n* = 6).

**TABLE 1:** Grouping and dosing schedule.

Group number	Group information	Drug concentration (mg/ml)	Injection volume (μL)	Number		Injection method	Autopsy (d)
				Male	Female		
G1	Control group	0	10	3	3	Intradisc injection, once	28
G2	Low dose group	0.1	10	3	3		28
G3	Middle dose group	1	10	3	3		28
G4	High dose group	10	10	3	3		28

### Surgical Methods and Simvastatin Treatment

The animal used in this experiment is a 6-month-old New Zealand male rabbit. At this time, the animal has matured (skeletal muscle, intervertebral disc, etc.), and it will not cause model failure or affect the experimental results due to development. All injectable drugs are administered intramuscularly (i.m.) during the perioperative period. Atropine (0.2 mg/kg) was administered preoperatively to reduce the production of bronchial secretions during surgery. After about 5–10 min, all animals were first anesthetized with Shutai (10–15 mg/kg, i.m.). During surgery, 1.5–3.0% isoflurane is used to maintain anesthesia, and the oxygen flow used is 0.8–1.5 L.

**FIGURE 1:**
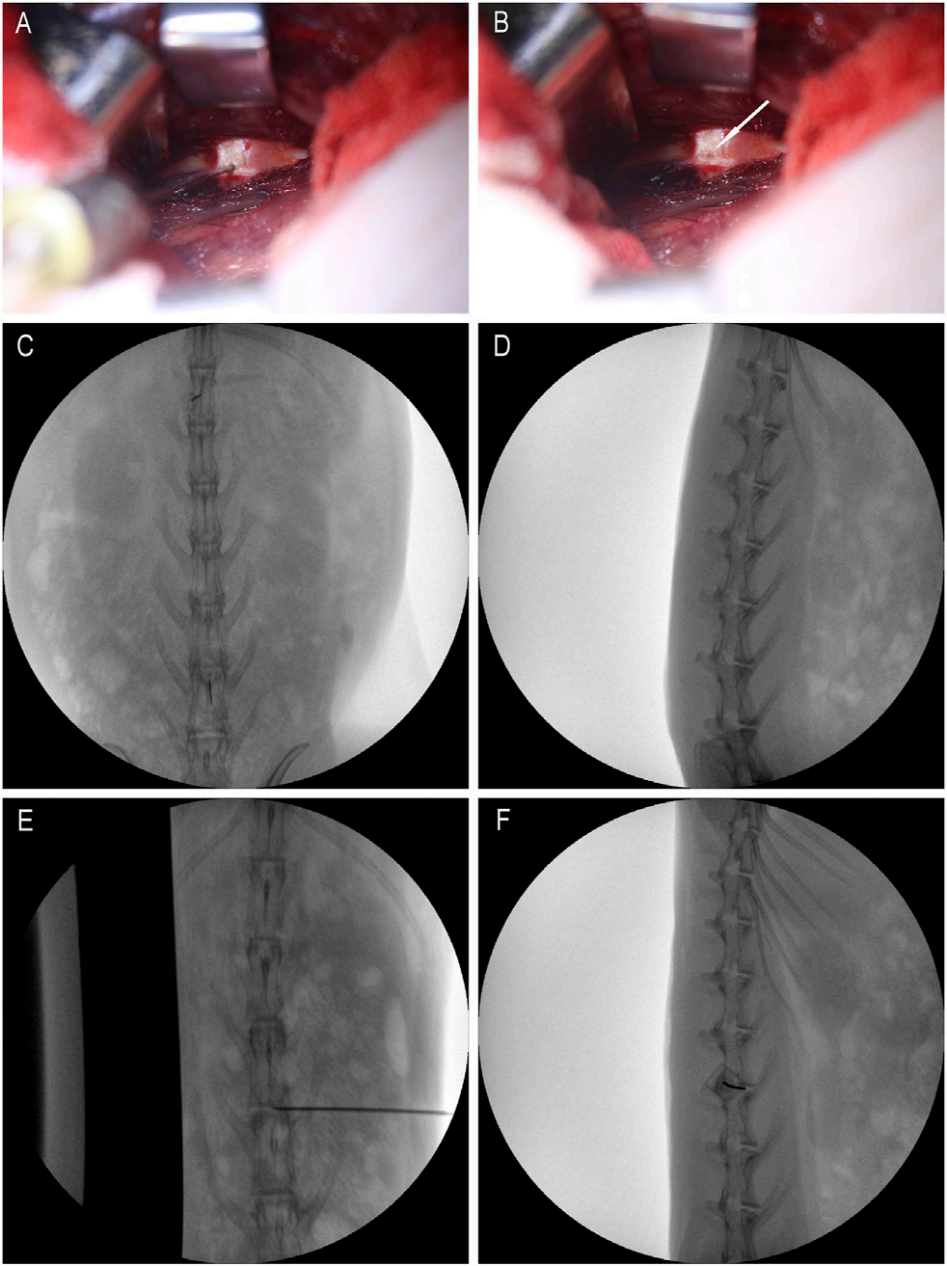
Intraoperative rabbit picture. **(A)** is the picture when the micro-syringe is injected; **(B)** is the picture when the micro-syringe is withdrawn; the white arrows are pinholes; **(C,D)** are positioning films before exposure; **(E,F)** are X-ray films during puncture.

After anesthesia, the rabbit was placed in the left lateral position, and the preoperative site was skinned. Sterilize the area to be punctured with iodophor and spread a towel. With the aid of a C-arm machine and a positioning needle, the position of the disc to be punctured is clarified. Make an incision of about 3 cm in length at 3/4 cm from the spinous process at L3/4, L4/5 and L5/6, and expose the L3/4, L4/5 and L5/6 intervertebral disc. Use a mini syringe to connect to a 23G needle to puncture the disc (Figure 1A). According to previous research, 23G needles do not cause disc degeneration. The puncture is performed under the guidance of X-rays (Figures 1E,F). The direction of the puncture needle is parallel to the upper and lower end plates. The depth of the puncture needle entering the disc is 5 mm with hemostats. The volume injected in each disc is 10 μL, and the injection time is 1 min. After the injection, maintaining the needle in the intervertebral disc for 1 min, slowly withdraw the puncture needle to prevent fluid leakage (Silveira et al., 2014). The administration schedule is shown in Table 1 Either PEG-PLGA-PEG gel loaded with 2 μL of simvastatin (LKT Laboratories, St. Paul, MN, United States) or gel alone was slowly injected into the discs.

### Animal Health Observation

The rabbit’s weight and food residue were recorded once a week.

### Pharmacokinetics and Toxicology

Blood samples were collected at the time points in Table 2 for pharmacokinetic and toxicity studies. Prior to rabbit surgery, a catheter was placed through a ear marginal vein. Approximately 300 μL of a blood sample was collected through a venous catheter. Blood samples were collected in tubes coated with lithium heparin and containing 100 μM semicarbazide. The samples were stored on ice and plasma was obtained by centrifugation (approximately 100 μL). All blood samples are centrifuged within 30 min of blood collection and plasma samples are obtained. The method of centrifugation is: centrifugation at a temperature of 4°C for 5 min at a speed of 3,000 g. Plasma samples were collected after centrifugation and transferred to labeled polypropylene tubes for PK analysis. Plasma samples were stored frozen at −80°C until further analysis.

**TABLE 2:** Blood collection time points.

Grouping	Number of animals	Blood collection time	Total number of specimens
G2 (Low dose group)	3	Preoperative, postoperative 6 h, 24 h, 3 d, 7 d, 14 d, 28 d	75 (42 + 33)
G4 (High dose group)	3		
G3 (Middle dose group)	3	Preoperative, postoperative 0.5 h, 1 h, 3 h, 5 h, 8 h, 24 h, 3 d, 7 d, 14 d, 28 d	

*The low dose group was injected with hydrogel + simvastatin (0.1 mg/ml), the medium dose group was injected with hydrogel + simvastatin (1 mg/ml) and the high dose group was injected with hydrogel + simvastatin (10 mg/ml).

Low-dose and high-dose groups: Blood samples were collected from the low-dose and high-dose groups of three male animals in each group and processed into plasma at seven time points (see Table 2).

Medium dose group: Blood samples were collected from three male animals and processed into plasma at 11 time points (see Table 2).

### Pharmacokinetic Analysis

Plasma samples were analyzed by liquid phase mass spectrometry (Supplementary Table 1). Determine pharmacokinetic parameters based on study-directed average concentration-time data in test drugs. The parameters (drug peak concentration, peak time and end elimination rate, etc.) were calculated using a non-isolated module from WinNonlin® Professional 6.3. Any BLQ (10 ng/ml) concentration was excluded from the calculation of the PK parameters Supplementary Table S1.

### Collection and Detection of Cerebrospinal Fluid

Three male rabbits were also exposed to the L3/4, L4/5, and L5/6 discs via a retroperitoneal approach. These discs were injected with 10 μL of high concentration simvastatin (10 mg/ml). At the same time, a subdural catheter was placed on the back of the rabbit to obtain 100 μL of cerebrospinal fluid before, 3, 5, 8, 24 h, 3, 7, 14 and 28 days after the operation. Cerebrospinal fluid-liquid phase mass spectrometry (Supplementary Table 2) was used to analyze cerebrospinal fluid samples.

### Clinical Blood and Urine Tests

On day 0 and day 28, blood and urine samples were collected from all rabbits, and blood routine, blood biochemical, and urine routines of all animals were analyzed and evaluated Supplementary Table S2.

### Tissue and Organ Evaluation and Weighing

28 days after the intravertebral disc was injected with drugs, rabbits were sacrificed by injecting an excessive amount of anesthetics, and autopsies were performed on all animals. Observe the general morphology of the adrenal gland, brain, heart, kidney, liver, ovary, pituitary, prostate, spleen, thyroid, parathyroid gland, thymus, testis and uterus of each animal and weigh them.

### Histological Assessment

Twenty-eight days after the disc was injected with the drug, rabbits were sacrificed by injection of an overdose of anesthetic, and all disc tissues between L3-L6 were collected. These tissues were fixed in a 10% formaldehyde solution for at least 48 h. They were then decalcified for 5–7 d in 22.5% formic acid and 10% sodium citrate. Decalcification is regularly verified using radiography. They were then paraffin-embedded and sagittal (10 μm thick) using a microtome. Sections were stained with hematoxylin and eosin (H&E) and then qualitatively analyzed using an Olympus light microscope at magnifications ranging from 40 to 200 times. Observe the histological HE staining results and score according to the evaluation method in Table 3.

**TABLE 3:** Histological rating scale.

Content	Grade	Grade Description
I. Annulus fibrosus	1	Normal pattern of fibrocartilage lamellae (U-shaped in the posterior aspect and slightly convex in the anterior aspect) without ruptured fibers and without a serpentine appearance anywhere within the annulus
	2	Ruptured or serpentine-patterned fibers in less than 30% of the annulus
	3	Ruptured or serpentine-patterned fibers in more than 30% of the annulus
II. Border between the annulus fibrosus and nucleus pulposus	1	Normal
	2	Minimally interrupted
	3	Moderate/severe interruption
III. Cellularity of the nucleus pulposus	1	Normal cellularity with large vacuoles in the gelatinous structure of the matrix
	2	Slight decrease in the number of cells and fewer vacuoles
	3	Moderate/severe decrease (>50%) in the number of cells and no vacuoles
IV. Matrix of the nucleus pulposus	1	Normal gelatinous appearance
	2	Slight condensation of the extracellular matrix
	3	Moderate/severe condensation of the extracellular matrix

*The histological rating scale is based on four types of degenerative changes, with scores ranging from four points for normal discs (1 point per class) to 12 points for severe disc degeneration (3 points per class).

### Statistical Analysis

Data analysis was performed using PASW Statistics 21.0 (SPSS Inc.) software and graphing was performed using GraphPad Prism 8 software. All experimental data are expressed as mean ± standard deviation. Comparisons between groups were performed using one-way variance for statistical analysis. If there are differences and a normal distribution is satisfied, Bonferroni's multiple comparison test is performed. If the normal distribution is not satisfied, use the Kruskal-Wallis H test. *p* < 0.05 means that the difference is significant.

## Results

### Effect of Intravertebral Disc Drugs on Animal Weight

**FIGURE 2:**
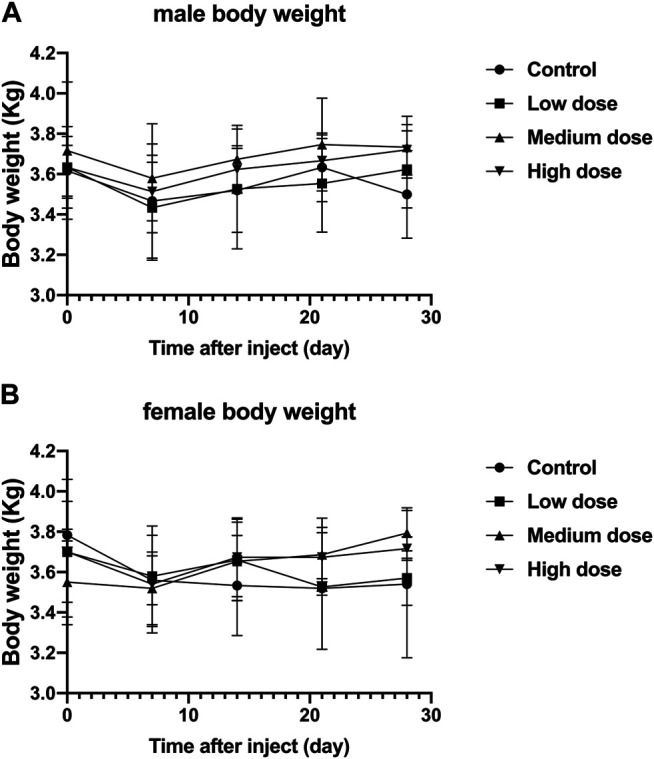
Effect of intra-vertebral disc injection of drugs on animal weight. **(A)** refers to the change in body weight of male rabbits in four groups over time. **(B)** refers to the change of body weight of female rabbits in four groups over time. All data are expressed as mean ± standard deviation (SD) (*n* = 3).

Regardless of male or female rabbits, all rabbits lost a little weight during the first 7 days after surgery, and then rose steadily. In male or female rabbits, there was no significant difference in body weight between the four groups of rabbits at each time point (*p* > 0.05, Figure 2A,B). The steady increase in body weight in all rabbits indicates that the animals are well tolerated for surgery and treatment.

### Effects of Intravertebral Disc Injections on Food Residues in Animals

**FIGURE 3:**
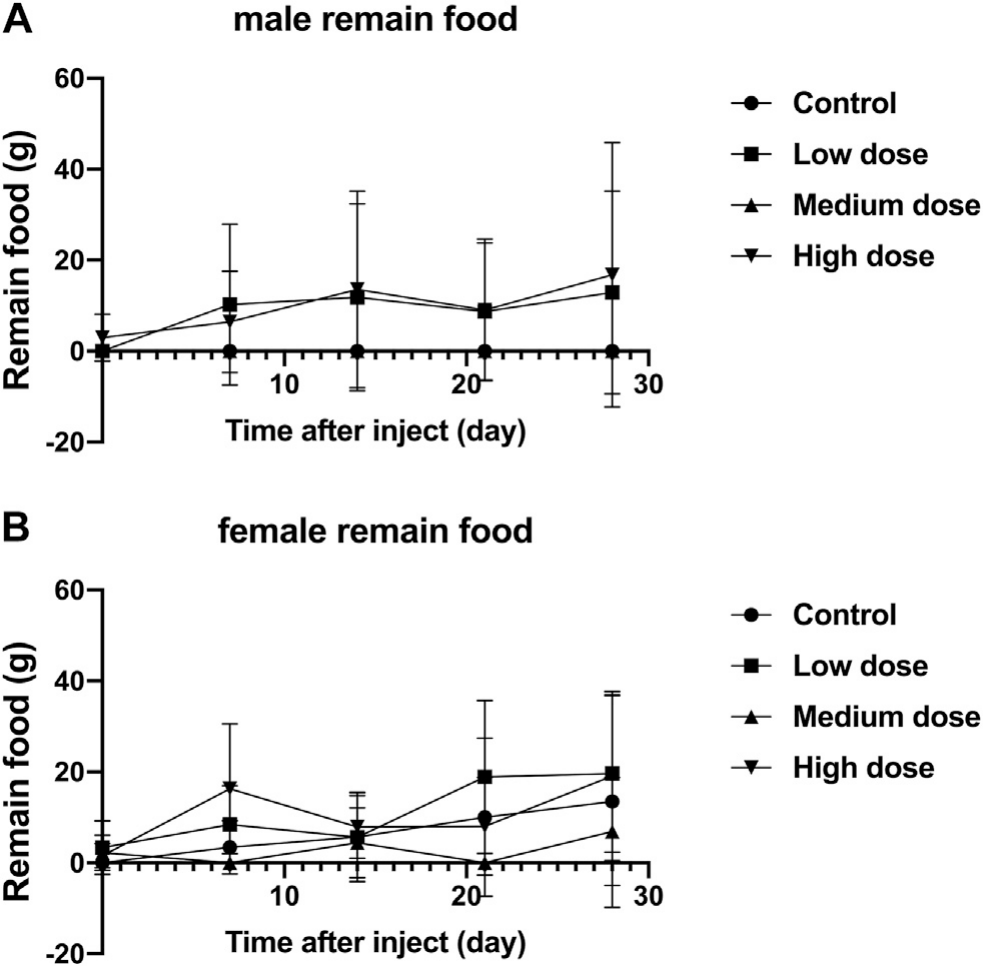
Effect of intra-vertebral disc injection of drugs on animal food residues. **(A)** refers to the change of food residues of male rabbits over time. **(B)** refers to the change of the food residue amount of female rabbits in four groups over time.

The food residues of all rabbits fluctuated with time, but there was no significant difference in the food residues between each group of animals (*p* > 0.05, Figure 3A,B). This shows that all rabbits can tolerate our surgery well and adapt to the relevant feeding environment.

### Effects of Intravertebral Disc Injections on Clinical Blood and Urine Tests in Animals

#### Blood Test

**FIGURE 4:**
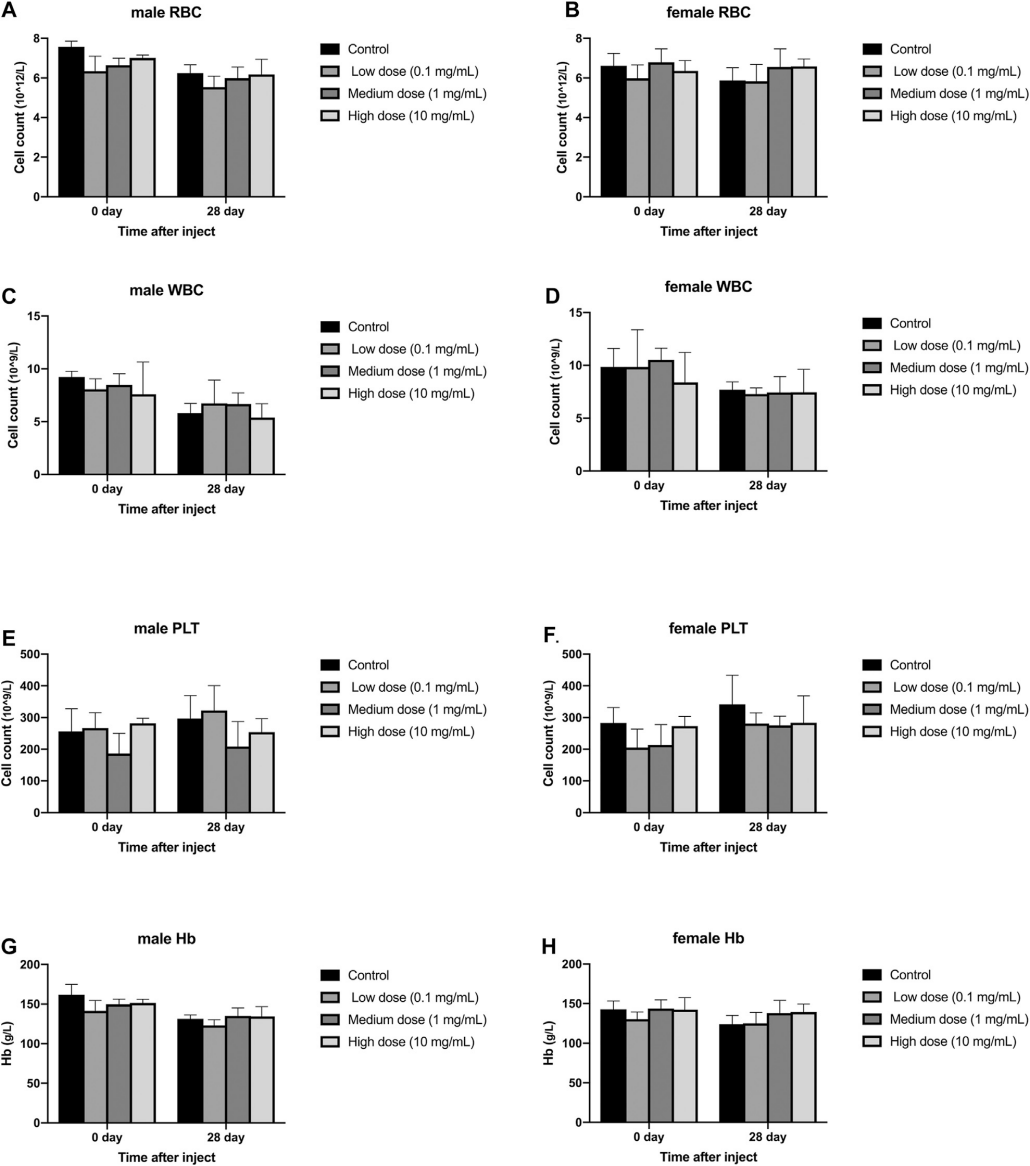
Effect of drug injection into the disc on blood routine of animals. **(A)** refers to the change of red blood cells of male rabbits over time. **(B)** refers to the change of red blood cells of female rabbits over time. **(C)** refers to the change of white blood cells of male rabbits over time. **(D)** refers to the change of the white blood cells of the female rabbits of the four groups over time. **(E)** refers to the change in platelets of male rabbits over time. **(F)** refers to the change in platelets of female rabbits over time. **(G)** refers to the change in hemoglobin of the four groups of male rabbits over time. H refers to the change of hemoglobin of the four groups of female rabbits over time. All data are expressed as mean ± SD (*n* = 3).

Different concentrations of simvastatin injected into rabbit intervertebral discs will not affect the blood routine of rabbits of different sexes. On days 0 and 28, blood samples from all animals were collected and tested by a blood analyzer. In male animals, the contents of red blood cells, white blood cells, and hemoglobin in the four groups were all lower than those on day 0, but the differences were not statistically significant (*p* > 0.05, Figure 4A,C,E); There was no significant difference between platelets on day 0 and day 28 (*p* > 0.05, Figure 4G). In female animals, there was no significant difference in the results of red blood cells, white blood cells, platelets, and hemoglobin on day 0 and day 28 (*p* > 0.05, Figure 4B,D,F). At day 0, there were no significant differences in the measurement results of red blood cells, white blood cells, platelets, and hemoglobin between the four groups in males and females (*p* > 0.05, Figure 4H). On the 28th day, in the measurement results of red blood cells, white blood cells, platelets, and hemoglobin, there was no significant difference between the four groups of male and female animals (*p* > 0.05, Figure 4A–H).

#### Blood Biochemical Examination

**FIGURE 5:**
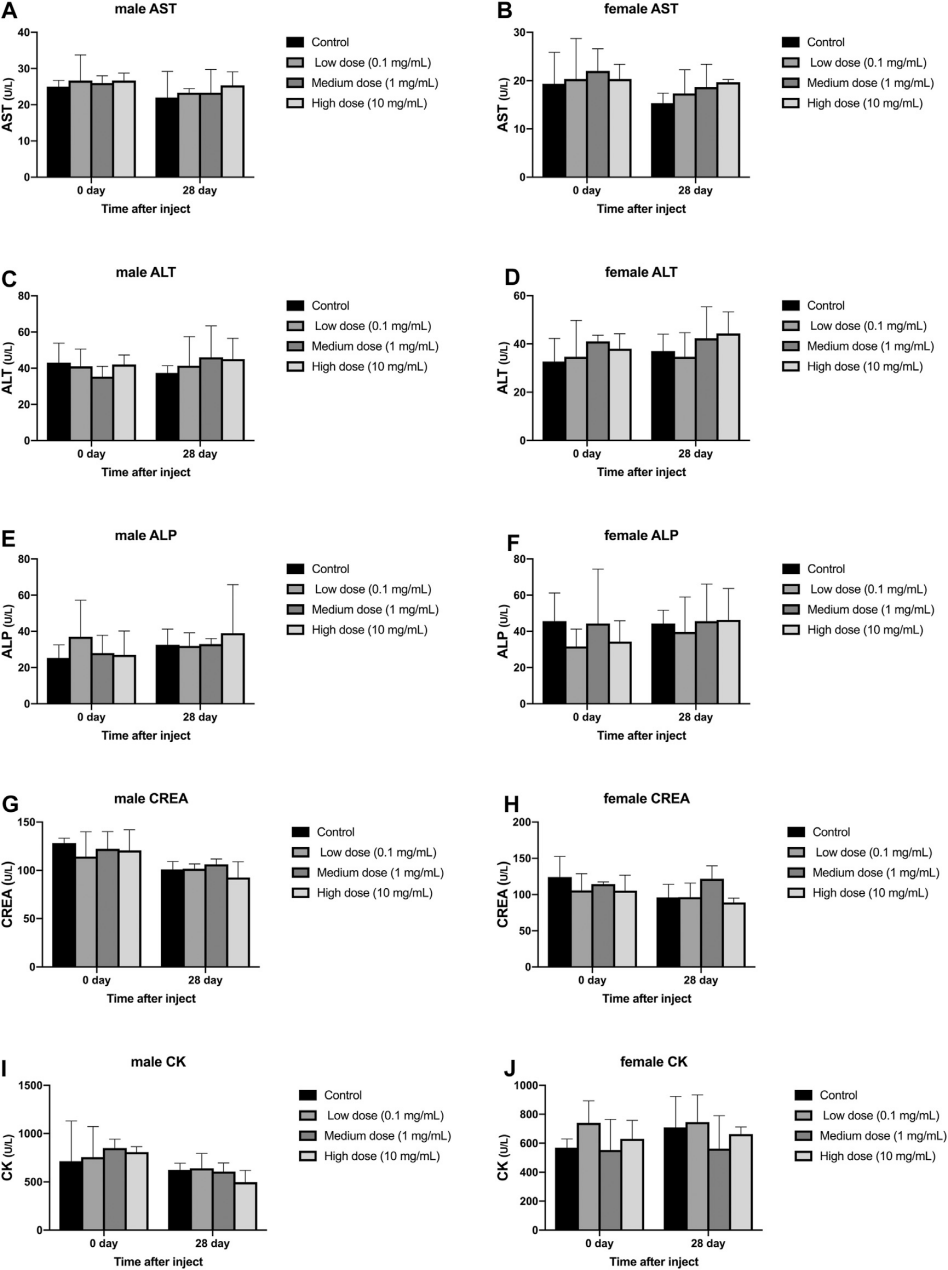
Effect of drug injection into the intervertebral disc on blood biochemistry of animals. **(A)** refers to the change over time of aspartate aminotransferase in four groups of male rabbits. **(B)** refers to the change of aspartate aminotransferase of female rabbits in four groups over time. **(C)** refers to the change of the alanine aminotransferase of the four groups of male rabbits over time. **(D)** refers to the change of alanine aminotransferase of female rabbits in four groups over time. **(E)** refers to the change of serum alkaline phosphatase over time in the four groups of male rabbits. **(F)** refers to the change of serum alkaline phosphatase over time in the four groups of female rabbits. **(G)** refers to the change of urea of the four groups of male rabbits over time. **(H)** refers to the change of urea of the four groups of female rabbits over time. **(I)** refers to the change of creatine kinase over time in the four groups of female rabbits. **(J)** refers to the change of creatine kinase of female rabbits in four groups over time. All data are expressed as mean ± SD (*n* = 3).

Different concentrations of simvastatin injected into rabbit intervertebral discs will not affect blood biochemistry of rabbits of different genders. On days 0 and 28, blood samples from all animals were collected and tested by an automatic blood biochemical analyzer. In male and female animals, there was no significant difference in serum aspartate aminotransferase, serum alanine aminotransferase, serum alkaline phosphatase, urea, and serum creatine kinase results on day 0 and day 28 (*p* > 0.05, Figure 5A–J). At day 0, the measurement results of serum aspartate aminotransferase, serum alanine aminotransferase, serum alkaline phosphatase, urea, and serum creatine kinase were not statistically significant between male and female animals (*p* > 0.05, Figure 5A–J). On the 28th day, the measurement results of serum aspartate aminotransferase, serum alanine aminotransferase, serum alkaline phosphatase, urea, and serum creatine kinase were not statistically significant between male and female animals (*p* > 0.05, Figure 5A–J).

#### Routine Urine Test

**FIGURE 6:**
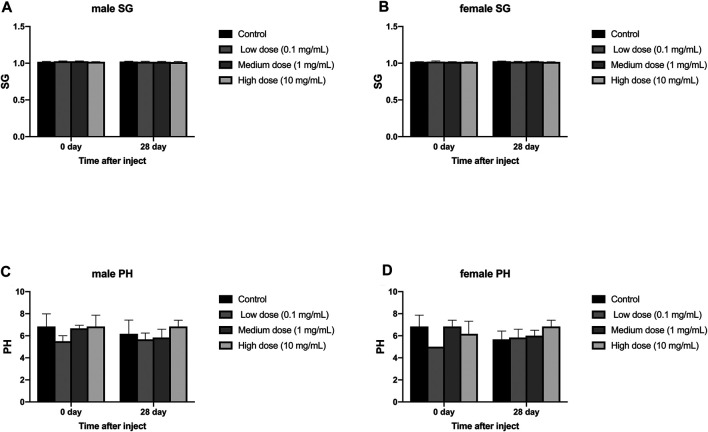
Effect of intra-disc injection of drugs on animal urine routine. **(A)** refers to the change in urine specific gravity of the four groups of male rabbits over time. **(B)** refers to the change in urine specific gravity of the four groups of female rabbits over time. **(C)** refers to the change over time of urine pH of the four groups of male rabbits. **(D)** refers to the change of urine pH value of female rabbits in four groups over time. All data are expressed as mean ± SD (*n* = 3).

The injection of different concentrations of simvastatin into the intervertebral disc of rabbits will not affect the urine routine of rabbits of different genders. On days 0 and 28, urine samples from all animals were collected and tested by an automatic urine analyzer. All rabbits showed yellow urine on day 0 and day 28, and no white blood cells, red blood cells, urine sugar, uroketoneuria protein, and urobilinogen were detected in urine. Only one rabbit had urinary bilirubin as a +. There was no significant difference in urine specific gravity and urine pH between day 0 and day 28 in the male and female groups (*p* > 0.05, Figure 6A–D). At day 0, in the measurement of urine specific gravity and urine pH, there were no significant differences between the four groups in males and females (*p* > 0.05, Figure 6A–D). On the 28th day, in the measurement of urine specific gravity and urine pH, there was no significant difference between the four groups in males and females (*p* > 0.05, Figure 6A–D).

### Effects of Drug Injection Into the Intervertebral Disc on Animal Pharmacokinetics

Blood was taken from different groups of animals at different time points (see the Tables 4, 5, 6) to obtain plasma, and the simvastatin content in the plasma was detected by liquid phase mass spectrometry. These results indicate that simvastatin was not detected in the blood in rabbits with different concentrations of simvastatin. (*p* < 0.05 represent statistically significant differences, *n* = 3).

**TABLE 4:** Simvastatin concentrations in plasma of G2 male rabbits after administration (ng/ml).

Time	Animal number			Mean	SD
	0911	0919	0921		
Pre-dose	BLQ	BLQ	BLQ	NA	NA
Post-6 h	BLQ	BLQ	BLQ	NA	NA
Post-24 h	BLQ	BLQ	BLQ	NA	NA
Post-3 D	BLQ	BLQ	BLQ	NA	NA
Post-7 D	BLQ	BLQ	BLQ	NA	NA
Post-14 D	BLQ	BLQ	BLQ	NA	NA
Post-28 D	BLQ	BLQ	BLQ	NA	NA

BLQ: Below the limit of quantitation. The data is expressed as the mean ± SD. (*n* = 3).

**TABLE 5:** Simvastatin concentrations in plasma of G4 male rabbits after administration (ng/ml).

Time	Animal number			Mean	SD
	0907	0908	0918		
Pre-dose	BLQ	BLQ	BLQ	NA	NA
Post-6 h	BLQ	BLQ	BLQ	NA	NA
Post-24 h	BLQ	BLQ	BLQ	NA	NA
Post-3 D	BLQ	BLQ	BLQ	NA	NA
Post-7 D	BLQ	BLQ	BLQ	NA	NA
Post-14 D	BLQ	BLQ	BLQ	NA	NA
Post-28 D	BLQ	BLQ	BLQ	NA	NA

BLQ, below the limit of quantitation. The data is expressed as the mean ± SD. (*n* = 3).

**TABLE 6:** Simvastatin concentrations in plasma of G3 male rabbits after administration (ng/ml).

Time	Animal number			Mean	SD
	0915	0916	0917		
Pre-dose	BLQ	BLQ	BLQ	NA	NA
Post-0.5 h	BLQ	BLQ	BLQ	NA	NA
Post-1 h	BLQ	BLQ	BLQ	NA	NA
Post-3 h	BLQ	BLQ	BLQ	NA	NA
Post-5 h	BLQ	BLQ	BLQ	NA	NA
Post-8 h	BLQ	BLQ	BLQ	NA	NA
Post-24 h	BLQ	BLQ	BLQ	NA	NA
Post-3 D	BLQ	BLQ	BLQ	NA	NA
Post-7 D	BLQ	BLQ	BLQ	NA	NA
Post-14 D	BLQ	BLQ	BLQ	NA	NA
Post-28 D	BLQ	BLQ	BLQ	NA	NA

BLQ, below the limit of quantitation. The data is expressed as the mean ± SD. (n = 3).

### Effects of Intravertebral Disc Injection of Drugs on the Morphology of Animal Tissues

Some animals show congenital abnormalities, such as no visible parathyroid tissue, large dilated cysts without fluid, and fatty tissue in the brain. No significant abnormality was observed in the organs of all animals, indicating that the experimental treatment scheme will not cause damage to various tissues and organs of the animals.

### Effect of Drug Injection iInto the Disc on Animal Organ Weight

Different concentrations of simvastatin injected into rabbit intervertebral discs will not affect the organ weight of rabbits of different genders. These organs include: adrenal, brain, heart, kidney, liver, ovary, pituitary, prostate, spleen, thyroid, parathyroid, thymus, testis, and uterus.

**FIGURE 7:**
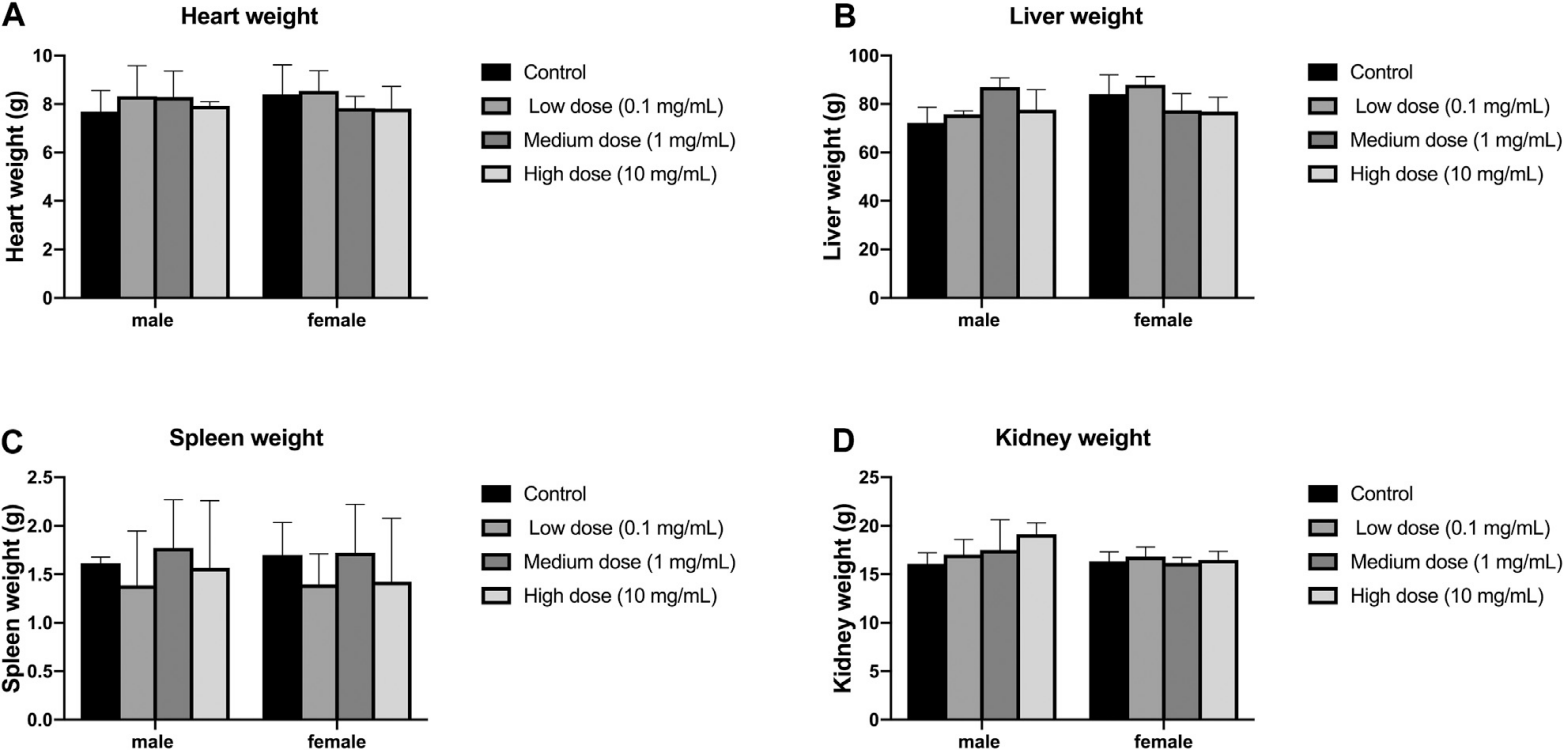
Effect of intra-vertebral disc injection on animal organ weight. **(A)** refers to the heart weight of four groups of rabbits of different genders. **(B)** refers to the liver weight of four groups of rabbits of different sexes. **(C)** refers to the spleen weight of rabbits of four different sexes. **(D)** refers to the kidney weight of four groups of rabbits of different genders. All data are expressed as mean ± SD (*n* = 3).

There was no significant difference in heart weight between the four groups of male and female animals (*p* > 0.05, Figure 7A); there was also no significant difference in heart weight between the groups of male and female animals (*p* > 0.05, Figure 7A). There was no significant difference in liver weight between the four groups of male and female animals (*p* > 0.05, Figure 7B); there was also no significant difference in the weight of each group between male and female animals (*p* > 0.05, Figure 7B). There was no significant difference in spleen weight between the four groups of male and female animals (*p* > 0.05, Figure 7C); there was also no significant difference in the weight of each group between male and female animals (*p* > 0.05, Figure 7C). There was no significant difference in kidney weight between the four groups of male and female animals (*p* > 0.05, Figure 7D); there was also no significant difference in kidney weight between the groups of male and female animals (*p* > 0.05, Figure 7D).

### Cerebrospinal Fluid Test After Intravertebral Disc Injection

**FIGURE 8:**
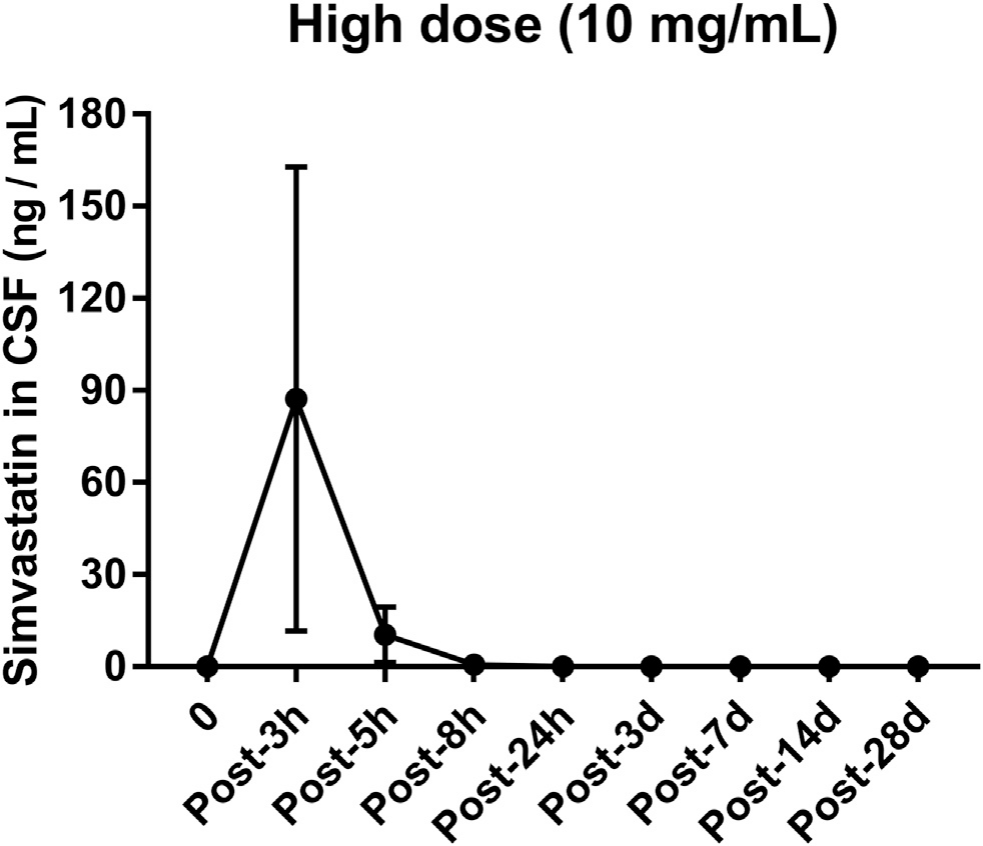
Changes in the concentration of simvastatin in the cerebrospinal fluid of patients after intra-disc injection of drugs over time. All data are expressed as mean ± SD (*n* = 3).

The concentration of simvastatin in the cerebrospinal fluid increased sharply after injection, reached a peak (133.8 ng/ml, Figure 8) 3 h after the operation, and fell outside the detectable range 24 h after the operation. From 1 to 28 d after surgery, the concentration of simvastatin in the cerebrospinal fluid was below the detectable range (Figure 8).

### Effects of Drugs Injected Into the Intervertebral Disc on the Intervertebral Disc

**FIGURE 9:**
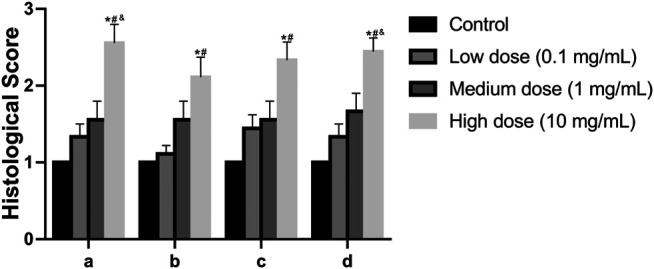
Effect of the drug on the disc after injection into the disc. **(A)**: Annulus fibrosus, **(B)**: Border between the annulus fibrosus and nucleus pulposus, **(C)**: Cellularity of the nucleus pulposus, **(D)**: Matrix of the nucleus pulposus. All data are expressed as mean ± SD, *p < 0.05 vs. vehicle control group; ^#^ *p* < 0.05 vs. low-dose group; ^&^ *p* < 0.05 vs. medium-dose group; (*n* = 3).

In the anulus fibrosus histological score, the score of the high-dose group was higher than that of the vehicle control group, the middle-dose group, and the low-dose group (*p* = 0.0009, *p* = 0.0347, *p* = 0.0404, Figure 9). In the histological score of the border between anulus fibrosus and the nucleus pulposus, the scores of the high-dose group were higher than those of the vehicle control group and the low-dose group (*p* = 0.0117, *p* = 0.0347, Figure 9). In the histological score of nucleus pulposus cell number and morphology, the scores of the high-dose group were higher than those of the vehicle control group and the low-dose group (*p* = 0.0021, *p* = 0.0373, Figure 9). In the histological score of the nucleus pulposus matrix, the scores of the high-dose group were higher than those of the vehicle control group, low-dose group, and middle-dose group (*p* = 0.0002, *p* = 0.0116, *p* = 0.0330, Figure 9). These results indicate that the low-dose group and the medium-dose group will not cause adverse effects on the intervertebral disc; at the same time, it also shows that the high-dose group will have a certain damaging effect on the local intervertebral disc.

## Discussion

In this study of pharmacokinetics and toxicology of simvastatin, we selected three drug concentrations. Choose high doses to show potential potentially adverse effects of the drug; choose low doses as the "clear" dose, which is the concentration of the drug that is cleared per unit of time; medium doses are the doses used to test the efficacy of the drug. According to the results of animal weight and animal food residues in the study, rabbits injected three doses of simvastatin can well tolerate surgery and simvastatin. The hemodynamic results show that the injected high dose of simvastatin is well metabolized and the blood concentration is below the measurable value. Observation of blood routine, blood biochemistry, urine routine, organ weight and morphology in this experiment showed that the three doses of simvastatin injected had no effect on the liver, kidney and hematopoietic system. The histological results of the intervertebral disc showed that the high-dose group would cause certain damage to the local disc tissue. The possible cause of local damage of high-dose simvastatin on intervertebral disc tissue is that local high-level simvastatin may have certain toxic effects on nucleus pulposus cells, fibroblasts and even chondrocytes. Cerebrospinal fluid is used to detect the leakage of drugs in surrounding tissues. The results of this study show that simvastatin appears in cerebrospinal fluid. The possible reason is that when simvastatin was injected, the puncture needle penetrated the entire disc and punctured the dural sac, so after the injection of the drug, the drug will enter the cerebrospinal fluid along the needle channel. Over time, the needle channel is closed, and the simvastatin content in the cerebrospinal fluid will disappear.

Simvastatin, a HMG-CoA reductase inhibitor, has been widely used clinically to regulate lipid metabolism and has great help in preventing/treating cardiovascular disease. It also has other effects: improving endothelial function, stabilizing atherosclerotic plaques, increasing nitric oxide (NO) synthesis, anti-inflammatory and anti-thrombotic effects. At present, oral simvastatin has been recognized to be very safe and reliable, but it is difficult to achieve the required drug concentration locally in the intervertebral disc when oral conventional doses of simvastatin are used. In clinical practical applications, when oral or systemic administration is used, the drug concentration required to stimulate bone and cartilage anabolic metabolism needs to be very high. Currently, the most reported adverse reactions with simvastatin are musculoskeletal reactions such as rhabdomyolysis, inflammatory myopathy, myasthenia gravis, Guillain-Barre syndrome, tendinopathy and amyotrophic lateral sclerosis (ALS) or ALS-like Syndrome has limited its clinical application (Golomb and Evans, 2008). This adverse reaction comes from the pleiotropic effects of statins and mitochondrial mechanisms. Consumption of isoprenoid compounds (the so-called "multiple action" effect of statins) results in a loss of protein isoprene and reduction of coenzyme Q-10, which promotes apoptosis, oxidation and permanent mitochondrial localization defect (Mammen and Amato, 2010). Uncommon adverse events include arthropathy, liver toxicity, insomnia, memory loss, insanity, peripheral neuropathy, erectile dysfunction, diabetes, impaired myocardial contractility, and autoimmune diseases (Godlee, 2014; Ray et al., 2014). Therefore, the method of local administration chosen in this study is to reduce the adverse effects of simvastatin on the system.

In addition, since the half-life of simvastatin is only 3 h, it is difficult to maintain the required drug concentration locally in the disc for a long time. Therefore, this study chose hydrogel triblock poly (ethylene glycol) -poly (lactic-co-glycolic acid) -poly (ethylene glycol) (PEG-PLGA-PEG) polymer as the drug carrier because it has the properties of injectability, temperature sensitivity, degradability, and biocompatibility have been proven to delay the release of drugs, thereby prolonging the action time of drugs (Chang et al., 2007; Tyagi et al., 2004; Zhang and Lin, 2008). Using PEG-PLGA-PEG hydrogel as a simvastatin transport vehicle minimizes the possibility of disc cells being exposed to transiently high concentrations of the drug during injection. This triblock polymer has been widely used as a vehicle for controlled drug or gene delivery (Jeong et al., 2000a; Li et al., 2003; Tyagi et al., 2004). Data from one study also demonstrated that the release profile of simvastatin in an *in vitro* PEG-PLGA-PEG gel was stable and sustainable. Some authors use the hydrogel formed by horseradish peroxidase (HRP) and hydrogen peroxide as a drug carrier (Than et al., 2014). In addition, polymers were selected for their sol-gel phase transition and slow degradation properties (Jeong et al., 2000b). After adding simvastatin to a liquid gel and injecting it into the disc, the compound quickly turns into a gel phase as soon as it reaches body temperature. When the gel was formed, only the IVD cells located in the center of the disc were exposed to simvastatin and it was sustained-released (Jeong et al., 1997). The gel is safer because PEG-PLGA-PEG hydrogels have been well used and validated, and a phase I clinical trial for breast cancer is currently underway (Vukelja et al., 2007).

There are some deficiencies in this experiment. First: The experimental object of this study is a New Zealand rabbit, which is still only a medium-sized animal, which is quite different from humans (different in mechanical structure and cell function), so we will choose animals that are closer to human characteristics in the next experiments or clinical trials in humans. Second: The duration of observation in this study is 28 days. It is possible that the residual toxicity or teratogenicity of some drugs has not yet appeared. Therefore, our future research will increase the observation time after injection of drugs. Third: This experiment is to observe the toxicity of simvastatin by injecting a hydrogel containing simvastatin into a normal disc. The ultimate goal of the experiment was to treat degenerative discs. The integrity, occlusion, osmotic pressure, and number of intervertebral disc cells of normal and degenerative discs are different, so they cannot truly simulate the actual clinical situation. Therefore, we will establish a disc degeneration model and then inject simvastatin to further study its toxicological effects.

## Conclusion

This experiment planned with the purpose to examine the 28-day safety of intra-disc injected once of simvastatin and the results showed no toxic signs from the delivered three dosages to three separate groups of males and females. The evaluations by body weight, food consumption, blood chemistry, complete blood count, gross observation at necropsy and microscopy histology revealed no abnormality related to the simvastatin.

## Data Availability

All data generated or analysed during this study are included in this published article.
