# Supplementary material for: Toxicology and Pharmacokinetics Study of Intradiscal Injection of Simvastatin in Rabbits
Source: Front Pharmacol. 2021 Apr 23;12:582309. doi: 10.3389/fphar.2021.582309 (PMC8103611; doi:10.3389/fphar.2021.582309)
Supplement: Supplementary file 1 [file Datasheet1.pdf]

Supplementary Table S1 : The plasma samples were analyzed by liquid phase mass spectrometry. The pharmacokinetic parameters are determined based on the average concentration-time data in the research-oriented test drug. Use WinNonlin® Professional 6.3 non-isolated module to calculate parameters (drug peak concentration, peak time and final elimination rate, etc.). The calculation of PK parameters does not include any BLQ (10ng/mL) concentration.

LC-MS methods

LC method (HPLC: Shimadzu LC20AD)				
Column:	Waters ACQUITY BEH C8 1.7um 2.1*50mm			
Mobile Phase:	A:	0.1% formic acid (FA) aqueous solution	B:	0.1% FA in acetonitrile (CAN)
HPLC Gradient				
Total time (min)		Flow rate (μL/min)	A (%)	B (%)
0.01		500	70	30
0.3		500	70	30
1.3		500	2	98
2		500	2	98
2.2		500	70	30
2.7		500	70	30
Mass Spectrometer Conditions (Mass Spectrometer: API4000)				
Ionization Mode:	ESI			
Mass Spectrometer Conditions:				
Compound ID:	Q1	Q3	DP	CE
Dic	296	250.1	50	17
Simvastatin-1	419.2	199.5	100	15
Sample processing:				
For rabbit plasma samples: In an EP tube, 5 μL of duplicate samples, QC samples and rabbit plasma samples with 150 μL ACN (50 ng / mL propranolol and 200 ng / mL tolbutamide and 500 ng / mL diisopropylcarbodiimide). Vortex the mixture for 1 minute, centrifuge for 10 minutes (13,000 rpm, 4 ° C), transfer 50 μL of the supernatant to a 96-well plate containing 150 μL of pure water, shake for 10 minutes, and finally inject 10 μL into the liquid phase. Grade mass spectrometry system.				

Supplementary Table S2 : Cerebrospinal fluid-liquid phase mass spectrometry. Rabbit cerebrospinal fluid before, 3h, 5h, 8h, 24h, 3, 7, 14 and 28 days after the operation were tested.

Cerebrospinal fluid-liquid phase mass spectrometry

HPLC:	Shimadzu LC20AD				
Autosampler:	CTC PAL				
Mass Spectrometer:	API4000				
LC Method					
Column:	Thermo Hypersil Gold 1.9um 50*2.1mm				
Mobile Phase:	A:	0.1%FA in water	B:	0.1%FA in ACN	
HPLC Gradient					
Total Time(min)	Flow Rate(μL/min)	A(%)	B(%)		
0.01	500	70	30		
0.30	500	70	30		
0.90	500	40	60		
2.00	500	5	95		
2.40	500	5	95		
2.50	500	70	30		
3.00	500	70	30		
Mass Spectrometer Conditions					
Ionization Mode:	ESI				
Mass Spectrometer Conditions:					
Compound ID:	Q1	Q3		DP	CE
Tol	271.0	74.2		70	22
Simvastatin-1	419.2	199.5		100	15
Sample processing:					

Rabbit cerebrospinal fluid samples: In an EP tube, duplicate samples of 10 μL, duplicate QC samples and rabbit cerebrospinal fluid samples with 60 μL ACN (50 ng/mL propranolol and 200 ng/mL tolbutamide and 500 ng/mL diisopropylcarbodiimide). Vortex the mixture for 1 minute, centrifuge for 10 minutes (13,000 rpm, 4 ° C), transfer 50 μL of the supernatant to a 96-well plate containing 150 μL of pure water, shake for 10 minutes, and finally inject 10 μL into the liquid phase. Grade mass spectrometry system.
